# Supplementary material for: When two are better than one: Modeling the mechanisms of antibody mixtures
Source: PLoS Comput Biol. 2020 May 4;16(5):e1007830. doi: 10.1371/journal.pcbi.1007830 (PMC7224563; doi:10.1371/journal.pcbi.1007830)
Supplement: Attachment — Submitted filename: 2020-01-24TE Reviewer Comments.pdf [file pcbi.1007830.s005.pdf]

Reviewer 1

A cocktail of antibodies can be a superior treatment in comparison to single monoclonal antibodies, but understanding the mechanisms behind this observation is still missing. The manuscript by Einav et al. proposes a framework for prediction of the activity of the mixture of antibodies based on measured parameters of individual antibodies (such as affinity, activity and epitope mapping), and, thus, the manuscript addresses an important question. Authors used several experimental data sets to verify their model predictions, and their analysis reports interesting results. Although the manuscript is well written, and I enjoyed reading it, it has some limitations that need to be addressed before its publication:

1. The model introduced the parameter α (in lines 48-49 and S1 text) ranging from 0-1, where $\alpha = 1$ corresponds to no effect and $\alpha = 0$ implies complete inhibition of receptor activity upon binding by antibody. The underlying model assumes the monotonic relationship between binding of EGFR receptor by growth factors and activation of the cells. No activation will be seen if all EGFR receptors are blocked by antibodies.

The problem appears when the parameter α is estimated in Figure S2. Parameter $\alpha < 1$ for all 10 antibodies was derived for HN5 line, but $\alpha > 1$ for three antibodies for A431NS. $\alpha > 1$ corresponds to the case where blocking of all EGFR receptors with antibodies will lead to maximal cell growth. Comparison of α values for HN5 and A431NS for these three antibodies suggests that the key hidden assumption of the model about monotonic relationship between binding of EGFR receptors by growth factors and activation of the cells is somehow not true (at least for these three antibodies and used concentrations).

Author Reply

We thank Reviewer 1 for raising this excellent point. While most antibodies would be expected to bind and reduce EGFR activity by inhibiting ligand binding, some antibodies may also increase EGFR activity (e.g. by stabilizing an EGFR conformation with high ligand affinity). An excellent example of work featuring both disease enhancing ($1 < \alpha$) and disease protection ($0 < \alpha < 1$) antibodies is Chow 2013, where they examined antibodies against the *Bacillus anthracis* protective antigen. (Multiple other papers featuring disease enhancing antibodies are cited in that same work.) Thus, we do not think that antibodies with potency parameters of $\alpha > 1$ indicate a problem, although we agree that this point should be clarified as shown in our revisions below.

Regarding the EGFR antibodies we analyzed, we used the following fractional activity equation,

$$\text{Fractional Activity} = \frac{1+\alpha\frac{c}{K_D}}{1+\frac{c}{K_D}},$$

which exhibits three different behaviors depending on whether $0 < \alpha < 1$, $\alpha = 1$, or $1 < \alpha$ (see Figure S1 below). Fractional activity monotonically decreases with the concentration c of antibody when $0 < \alpha < 1$ and monotonically increases when $1 < \alpha$. We now explicitly discuss this fact in our revised description of α.

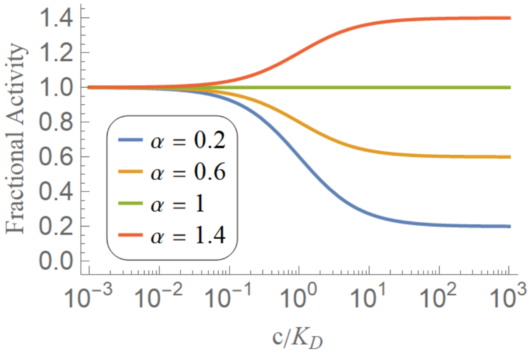

Figure S1. Fractional activity for different values of the potency parameter α. For convenience, the x-axis plots the concentration c divided by the dissociation constant K_D to make it dimensionless.

Lastly, it is worth noting that we propose no relationship between the α parameters for the HN5 and A431NS cell lines. Indeed, in the original work of Koefoed 2011, they showed that the rank-order of antibody mixtures in

the A431NS cell line (shown in Figure S2 below) does not correspond to the rank order of these mixtures in the HN5 cell line. Several of the antibody mixtures are enhancing in the A431NS cell line (values >100) and protective in the HN5 cell line (values <100). Our own analysis is in line with these data.

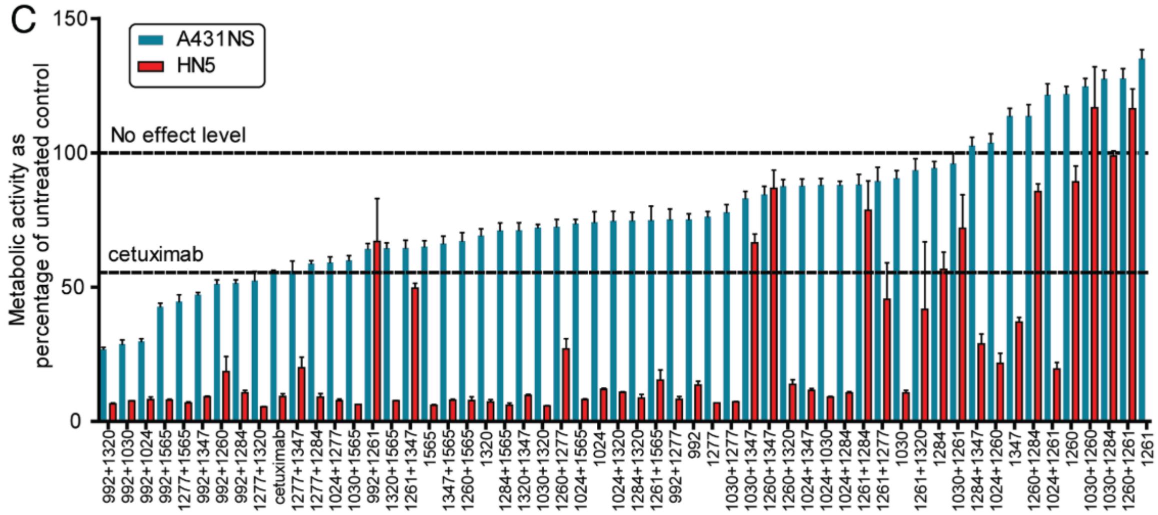

Figure S2. The fractional activity of different antibody mixtures (with total concentration of 2 μg/mL) in the A431NS (teal) and HN5 (red) cell lines. Figure taken directly from Koefoed 2011 Figure 2C.

To address the points above, we have made the following changes in our manuscript:

Old version (Page 4)

A value of $\alpha = 1$ represents an impotent antibody that does not affect activity while $\alpha = 0$ implies that an antibody fully inhibits activity upon binding.

New version (Page 4)

A value of $\alpha = 1$ represents an impotent antibody that does not affect activity while $\alpha = 0$ implies that an antibody fully inhibits activity upon binding. Antibodies with an intermediate value ($0 < \alpha < 1$) will partially inhibit receptor activity upon binding [Falkowska 2014], whereas antibodies with potency greater than one ($1 < \alpha$) will increase activity upon binding [Chow 2013].

Old version (Page S4)

Using the additional measurement of each antibody's potency at 2 μg/mL in the A431NS cell line, we can infer the potency α of each antibody in the A431NS cell line.

New version (Page S4)

Using the additional measurement of each antibody's potency at 2 μg/mL in the A431NS cell line, we can infer the potency α of each antibody in the A431NS cell line. We propose no relationship between the α parameters in the HN5 and A431NS cell lines, and as noted by Koefoed et al., some antibody mixtures may decrease activity in one cell line ($0 < \alpha < 1$) but increase activity ($1 < \alpha$) in the other cell line.

[Continued from above:] One way to deal with it is to analyze only 7 antibodies. Will it lead to better overall predictions in Figure 2C? Currently, model predicts higher fractional activity than measured for many antibody mixtures. Will it still be true if only 7 antibodies and their mixtures will be used in the analysis?

Author Reply

Indeed, our analysis does turn out to look significantly better for the 7 antibodies with potency less than 1. In fact, this turned out to be a key insight that we needed to make sense of the continuum model recommended by Reviewer 2’s Comment #2 (and we elaborate more on this point in that comment below). We are greatly indebted to both Reviewers for pointing out this intriguing part of the data.

2. What does R^2 in Figure 2B-D correspond to? Is it for comparison of the model predictions to the data (shown line $y = x$) or for the overall best fit of the data ($y = ax + b$, which is a different case and, in this case, it will be interesting to see parameters a and b)?

Author Reply

The R^2 values in Figure 2B-D (and in all other plots in our manuscript) compare the experimental data to the model prediction ($y = x$), and we have clarified this point in the Methods. While some groups may choose to compute R^2 relative to $y = ax + b$, we feel that in our work which emphasizes the predictive power of our model, fitting to $y = x$ represents the best theory-experiment comparison.

Old version (Page 18, Methods)

The coefficient of determination used to quantify how well the theoretical predictions matched the experimental measurements (Fig 2B-D, Fig 3B, Fig 4D) was calculated using…

New version (Page 18, Methods)

The coefficient of determination used to quantify how well the theoretical predictions matched the experimental measurements (relative to the dashed line $y = x$ in Fig 2B-D, Fig 3B, Fig 4D) was calculated using…

3. Please include the original experimental data as requested by the journal policy (for example, measured values for fractional activity data for Figure 2B) in the supplemental information

Author Reply

We have fixed this oversight in our new submission. S1 ZIP File now contains all of the experimental data, whereas S2 ZIP File contains a Mathematica notebook reproducing every plot in the manuscript.

Minor points:
1. p.4 line 72 “and their epitopes are mapped” add “by pairwise interactions”
2. suggestion for Figure 2B -- to change “2/3Abs” (it reads “two thirds of Abs” which is confusing) to “2Abs/3Abs” and additionally define circles and triangles somewhere in the text
3. few typos: see lines 146, 220 in main text and first sentence on p. S2

Author Reply

We thank Reviewer 1 for their close reading. The first two points have been implemented as suggested. We fixed the typo on page S2, but we did not find any errors in lines 146 and 220 and left them unchanged.

Reviewer 2

This paper addresses the timely topic of complex antibody-antigen interactions mediated by two or more antibodies simultaneously present. The authors take a clear biophysical modelling approach to predict the binding activity of antibody mixtures to EGFR and show that the model using epitope mapping data indeed improves upon models assuming only independent or competitive binding. I have the following observations:

1. The basic modelling approach is not new. A model introduced by Wagh et al., PLoS Pathogens, 2016 also considers competitive and independent binding and seems to have large overlap. Can the authors comment on how their model is different?

Author Reply

We agree that the competitive and independent binding models were introduced in the excellent work of Wagh 2016 in the context of HIV, and that both models are straightforward to derive from mass-action kinetics (indeed, we see this simplicity as a great strength of our model). The novelty of our approach comes from the two ways that we extend these models:

- Classifying antibody epitopes via these models: Knowing whether multiple antibodies bind to distinct or overlapping epitopes is necessary to predict their effectiveness when mixed together. Antibodies extracted from polyclonal serum are routinely characterized in terms of their binding affinity and potency, but their epitopes may not be mapped onto the antigen (e.g. Yewdell 1986, Lee 2019). Our work shows that in such cases, the antibody epitopes can be inferred via these models.
- Generalizing to multidomain antibodies: As demonstrated by the impressive array of antibodies in Spiess 2015 (a snippet of which is shown in Figure S3 below), immense effort has gone into designing new antibodies for potential immunotherapies. Many of these constructs tether multiple antibody domains together, and it remains an open question as to how such constructs should be assessed. For example, if the "TandAb" antibody in the top-left of Figure S3 is compared against a 1:1 mixture of the Fabs that it is composed of, we would expect the "TandAb" would have higher affinity due to avidity. Yet it is hard to predict how large of an effect this avidity will have, or to predict the effectiveness of other "TandAb" antibodies comprised of different Fabs. Our work can answer precisely these questions in this exciting field, and our assessment of Laursen 2018 shows how the competitive and independent binding models can be generalized to such contexts and the types of insights such analysis brings (e.g. predicting combinations of multidomain antibodies, demonstrating that their IgG scaffold shown in our Figure 5C,D only bound with one arm).

That said, we agree wholeheartedly with Reviewer 2's Comment #2 below, and we have added a section to characterizing the continuum of behaviors between the competitive and independent binding models, which adds yet another novel component to our work. Moreover, we have added citations to Wagh 2016 as well as Kong 2015 and Palmer 2017 when introducing the competitive and independent binding models to provide better context for where such models have been used.

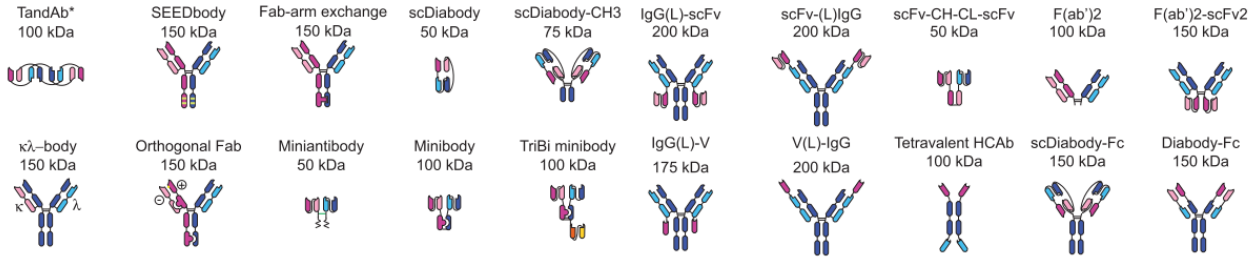

Figure S3. A sample of synthetic multidomain antibodies. Figure copied from Figure 1 of Spiess 2015.

2. The authors mention possible avenues to extend the basic model for antibody binding. In particular, synergy between antibodies can boost their activity with 10% and note that a more 'nuanced level of interaction' between antibodies could lead to a more precise prediction of dissociation constants. An extension of the binding model is, however, not pursued. In view of comment 1, I feel that an attempt to include 'a continuum of

possible interactions' to the binding model could greatly improve the novelty of the modelling approach taken by the authors.

Author Reply

We thank Reviewer 2 for raising this excellent point, and we have generalized our model to a continuum approach, where an antibody pair may now partially inhibit one another's binding by decreasing the Boltzmann weight of the doubly bound state in 1C (described in detail below). Interestingly, we first found that the resulting model performed *worse* ($R^2 = 0.87$) than our original model ($R^2 = 0.90$; where each antibody pair is characterized as either binding completely independently or completely competitively).

Harkening back to Reviewer 1's Comment #1, the issue turned out to be with the antibody mixtures containing Antibodies #8-10, which were the three antibodies that individually with potency $\alpha > 1$ that increased activity of EGFR. As explained below, once we accounted for this behavior, the continuum model showed a marked improvement over the original independent/overlapping model we had used.

We have added a new section to the main text to discuss this continuum model and summarize its key points. We have also added an SI section (reproduced below) that lays out this model in full detail. We thank both Reviewers for raising these excellent points, and we think the paper is much stronger for it!

New Version (Page S8, SI Section A.7)

Using the SPR measurements of the percent binding inhibition between each antibody pair, we defined the fraction $f = 1 - \frac{\%\ \text{inhibition}}{100}$ of the doubly bound EGFR state for each antibody pair. For example, Abs #1 and #3 inhibit each other's binding by 100% ($f = 0$) whereas Abs #2 and #3 inhibit each other by only 90% ($f = 0.1$). This fraction f multiplies the Boltzmann weight of the doubly bound state ($K_{D,\text{eff}}^{(2)} = K_D^{(2)}/f$ in Fig 1C), resulting in

$$\text{Fractional Activity} = \frac{1 + \alpha_1 \frac{c_1}{K_D^{(1)}} + \alpha_2 \frac{c_2}{K_D^{(2)}} + \alpha_1 \alpha_2 f \frac{c_1}{K_D^{(1)}} \frac{c_2}{K_D^{(2)}}}{1 + \frac{c_1}{K_D^{(1)}} + \frac{c_2}{K_D^{(2)}} + f \frac{c_1}{K_D^{(1)}} \frac{c_2}{K_D^{(2)}}}$$

for a 2-Ab mixture. Without synergistic interactions, f should lie between 0 and 1. Thus we assume that all values beyond this range are due to error and replace lower values with 0 and larger values with 1.

For 3-Ab mixtures, we assume that whenever any pair of antibodies (Abs j and k) are simultaneously bound, they diminish the statistical weight of that state by f_{jk}. This yields the relation

$$\text{Fractional Activity} = \frac{1 + \alpha_1 \frac{c_1}{K_D^{(1)}} + \alpha_2 \frac{c_2}{K_D^{(2)}} + \alpha_3 \frac{c_3}{K_D^{(3)}} + \alpha_1 \alpha_2 f_{12} \frac{c_1}{K_D^{(1)}} \frac{c_2}{K_D^{(2)}} + \alpha_2 \alpha_3 f_{23} \frac{c_2}{K_D^{(2)}} \frac{c_3}{K_D^{(3)}} + \alpha_1 \alpha_3 f_{13} \frac{c_1}{K_D^{(1)}} \frac{c_3}{K_D^{(3)}} + \alpha_1 \alpha_2 \alpha_3 f_{12} f_{23} f_{13} \frac{c_1}{K_D^{(1)}} \frac{c_2}{K_D^{(2)}} \frac{c_3}{K_D^{(3)}}}{1 + \alpha_1 \frac{c_1}{K_D^{(1)}} + \alpha_2 \frac{c_2}{K_D^{(2)}} + \alpha_3 \frac{c_3}{K_D^{(3)}} + f_{12} \frac{c_1}{K_D^{(1)}} \frac{c_2}{K_D^{(2)}} + f_{23} \frac{c_2}{K_D^{(2)}} \frac{c_3}{K_D^{(3)}} + f_{13} \frac{c_1}{K_D^{(1)}} \frac{c_3}{K_D^{(3)}} + f_{12} f_{23} f_{13} \frac{c_1}{K_D^{(1)}} \frac{c_2}{K_D^{(2)}} \frac{c_3}{K_D^{(3)}}}$$

where the triply bound state is maximally diminished by the multiplicative fractions $f_{12} f_{23} f_{13}$. Note that in the cases where all antibody pairs either bind purely competitively or independently ($f_{jk} \in \{0,1\}$), this equation reduces to the appropriate form of fractional activity given by Eqs (S6)-(S8).

Like the purely independent/competitive model of antibody binding, the continuum model can predict the fractional activity of each antibody mixture *a priori* from the individual monoclonal antibody dose-response curves and the SPR blocking data. However, plotting the resulting model predictions (Fig S4A; $R^2 = 0.87$) resulted in poorer predictions than the original independent/competitive model (Fig 2B; $R^2 = 0.90$).

More precisely, mixtures containing only Abs #1-7 matched the model predictions far better (Fig S4B; $R^2 = 0.94$) than mixtures containing Abs #8, #9, or #10 (Fig S4C; $R^2 = 0.84$). Notably, Abs #8-10 were the only antibodies that individually increased activity, yet when they were combined with other antibodies they decreased EGFR activity. For example, while Abs #8 and #10 individually increase activity by 1.14 and 1.35, respectively, their mixture decreases activity to 0.65. This suggested that when Abs #8-10 are simultaneously bound with another antibody, the mechanism of action by which they increase activity may be disrupted. This

idea is corroborated by the observation that antibody mixtures containing Abs #8-10 were systematically higher than the measured activity (Fig S4C).

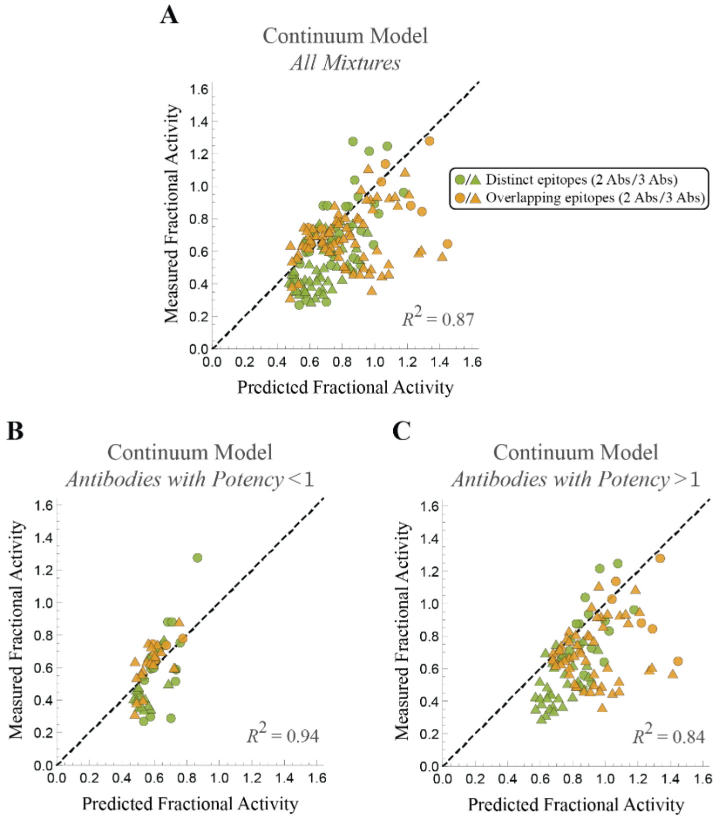

Figure S4. The continuum model for antibodies with potency less than or greater than one. (A) The predictions of the continuum model from Eq (S9) and (S10) for all antibody mixtures. (B) Predictions for mixtures containing antibodies with potency less than one (i.e. mixtures composed only of Abs #1-7). (C) Predictions for mixtures containing at least one antibody with potency greater than one (i.e. mixtures must contain either Ab #8, #9, or #10 together with any other antibodies).

To account for this behavior, we modified the continuum model so that when two antibodies are simultaneously bound (as in the final state in Fig 1C), their potencies are modified to the form $\alpha_{j.\text{eff}}$ (with one parameter per antibody, distinguished by the j subscript). With this modification, the continuum model for 2-Ab mixtures becomes

$$\text{Fractional Activity} = \frac{1 + \alpha_1 \frac{c_1}{K_D^{(1)}} + \alpha_2 \frac{c_2}{K_D^{(2)}} + \alpha_{1,\text{eff}}\alpha_{2,\text{eff}} f \frac{c_1}{K_D^{(1)}} \frac{c_2}{K_D^{(2)}}}{1 + \frac{c_1}{K_D^{(1)}} + \frac{c_2}{K_D^{(2)}} + f \frac{c_1}{K_D^{(1)}} \frac{c_2}{K_D^{(2)}}}$$

Similarly, all terms in the 3-Ab mixture equation with products of two or more α_j become $\alpha_{j.\text{eff}}$.

Whereas the original continuum model required no fit parameters, the $\alpha_{j.\text{eff}}$ parameters must be inferred from the antibody mixture data. As a first step, we used the original potency parameters $\alpha_{j.\text{eff}} = \alpha_j$ for the first seven antibodies ($1 \leq j \leq 7$) and only fit the $\alpha_{j.\text{eff}}$ parameters for Abs #8-10. This led to a marked improvement in the model's ability to characterize the antibody mixtures (Fig S5B, $R^2 = 0.95$) compared to when no effective potency parameters were applied (Fig S5A, $R^2 = 0.87$). The model predictions can be further improved if an effective potency parameter $\alpha_{j.\text{eff}}$ is fit for all ten antibodies (Fig S5C, $R^2 = 0.98$), but only the effective potency of Abs #3 and #4 change by more than 15% from their individual potency values (Fig S5G), suggesting that the

majority of these antibodies operate the same when individually bound to EGFR as when they are simultaneously bound with another antibody.

Lastly, we compared the continuum model against the original independent/competitive model by introducing the analogous $\alpha_{j,\text{eff}}$ parameters and characterizing each model's ability to characterize the activity of all antibody mixtures. While the continuum model with no effective potency parameters performed worse than the independent/competitive model (Fig S5D, $R^2 = 0.90$), the continuum model performed better when effective potency parameters were introduced for Abs #8-10 (Fig S5E, $R^2 = 0.94$) or for all ten antibodies (Fig S5F, $R^2 = 0.96$). Taken together, these results suggest that incorporating the spectrum of competition between antibodies can improve the ability to characterize antibody mixtures, and that antibodies (especially those that enhance EGFR activity) may behave differently when bound in combination with another antibody than when alone.

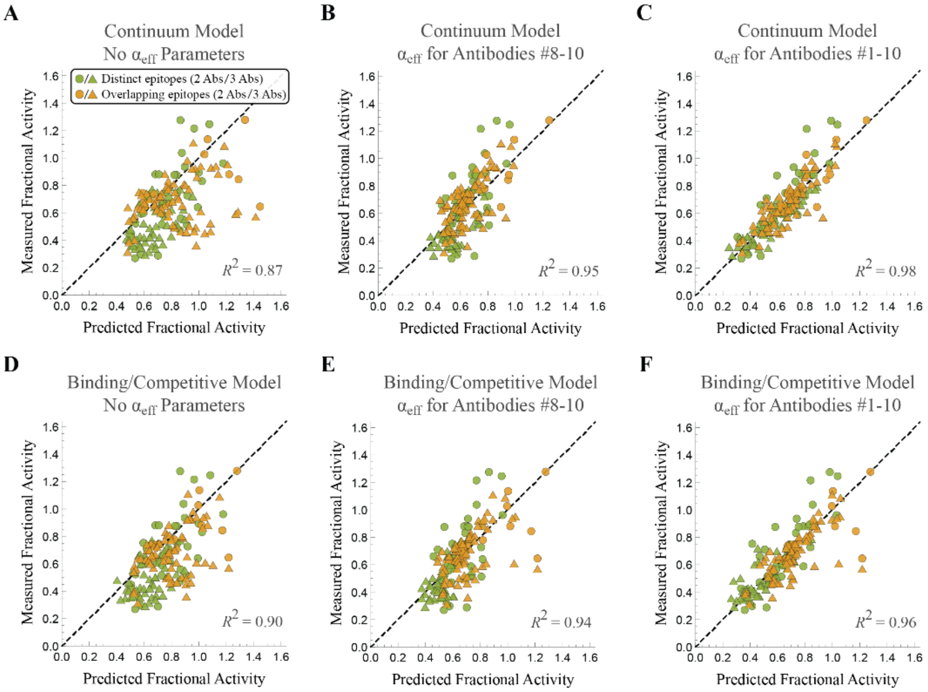

G

Antibody Index	α (Panel A)	α (Panel B)	α (Panel C)	α (Panel D)	α (Panel E)	α (Panel F)
Ab_1	0.65	0.65	0.68	0.65	0.65	0.65
Ab_2	0.69	0.69	0.76	0.69	0.69	0.70
Ab_3	0.74	0.74	0.86	0.74	0.74	0.81
Ab_4	0.73	0.73	0.36	0.73	0.73	0.43
Ab_5	0.75	0.75	0.71	0.75	0.75	0.76
Ab_6	0.90	0.90	1.00	0.90	0.90	0.98
Ab_7	0.94	0.94	1.01	0.94	0.94	1.02
Ab_8	1.16	0.60	0.60	1.16	0.60	0.62
Ab_9	1.26	1.03	1.07	1.26	1.03	1.09
Ab_{10}	1.44	0.97	0.95	1.44	1.02	1.06

Figure S5. Comparing different modeling schemes for the 2-Ab and 3-Ab mixtures. (A-C) The continuum model given by Eqs (S9) and (S10) where pairs of antibodies can partially inhibit one another's binding. (A) Using the potency parameters derived from monoclonal antibody dose-response curves Fig S2B). (B) Using effective potency parameters $\alpha_{j,\text{eff}}$ for antibodies #8-10 when they simultaneously bind with another antibody. (C) Using effective potency parameters for all ten antibodies when they simultaneously bind with another antibody. (D-F) Analogous plots using the original binding model from Fig 2B where each antibody pair binds purely independently or competitively. (G) The resulting potency parameters used in each scheme.

3. Another important point of the manuscript is that multidomain antibodies boost breadth and potency via avidity. However, avidity and boost free energies are not clearly integrated into the basic biophysical model and the corresponding discussion remains somewhat imprecise. I would suggest integrating the notion of avidity already at the level of equations (2) and (3).

Author Reply

We wholeheartedly agree with the spirit of this suggestion, and we believe that Appendix C.1 contained precisely this derivation (deriving the fractional activity of multidomain antibodies from the binding probability). This derivation introduces a single parameter ($\tilde{c}_{\text{eff}}$) to quantify the avidity of two tethered domains and demonstrates how this avidity parameter gets rescaled by the number of influenza hemagglutinin that must be bound to neutralize a virion.

To make this derivation more visible to the reader, we have added another section to the Methods titled "Models for influenza multidomain antibodies" that outlines this derivation and points to this SI section for a full explanation.

New Version (Page 22, Methods section called *Models for influenza multidomain antibodies*)

Influenza multidomain antibodies from Ref. [Laursen 2018] were characterized using a neutralization model derived in S1 Text Section C, Eq (S17). Combining a binding model that accounts for the avidity of the multiple domains together with a sigmoidal relationship between binding and neutralization [Ndifon 2009], we derive an expression for the neutralization of these multidomain antibodies. Assuming a Hill coefficient of 1 between binding and neutralization, this model is identical to the distinct binding model used for EGFR antibodies (1-Fractional Activity in Eq (2)) with potency $\alpha_j = 0$ for each antibody domain, $K_D \rightarrow \text{IC}_{50}$, and $c_2 \rightarrow c_{\text{eff}}$.

4. The authors motivate their study of antibody mixtures by stating that 'mounting evidence suggests that mixtures of antibodies can behave in fundamentally different ways [2]'. Does this refer to the competitive or independent binding dynamics of mixtures? The clinical trials that are mentioned in this reference only investigate bnAb-combinations with non-overlapping target sites, such that independent binding dynamics apply.

Author Reply

We thank the reviewer for raising this point. The reference to Caskey 2019 at the beginning of our introduction is intended to introduce important points about monoclonal and polyclonal antibody therapies that go beyond whether antibodies bind to the overlapping or distinct epitopes. Instead, it paints a broader picture of the mechanisms of action of polyclonal immunity that includes the following points:

- The clinical potential of a monoclonal or polyclonal antibody treatment against HIV-1 depends on the breadth, potency, and half-life of these antibodies. Our EGFR analysis deals with increased potency while the multidomain influenza antibodies we examine look at both breadth and potency.
- Polyclonal antibodies can reduce the possibility of escape mutations (as seen in both HIV-1 and cancer treatments) to better control the disease.
- Polyclonal or bifunctional antibodies can interact through their effector functions to elicit greater protection than would be available by individual antibodies.

We agree that these points were unclear as written, and the new version below is more precise.

Old version (Page 2)

While most therapies currently use monoclonal antibodies (mAbs), mounting evidence suggests that mixtures of antibodies can behave in fundamentally different ways [Caskey 2019].

New version (Page 2)

While most therapies currently use monoclonal antibodies (mAbs), mounting evidence suggests that mixtures of antibodies can lead to better control through improved breadth, potency, and effector functions [Caskey 2019].

5. From Fig. 5 on the prediction of IC50's of multi-domain antibodies, it is difficult to quantify the prediction of the model. Can the authors quantify how well you can predict the IC50 of the multi domain antibodies, and how much the model improves on an uninformed binding model?

Author Reply

We appreciate the Reviewer raising this point. We purposefully chose the style of Figure 5 of our main text (now Figure 6 in the new submission) to be different from the other plots in order to visually differentiate the multidomain flu antibody data from the EGFR analysis. However, we agree that it would also be helpful to include a standard plot where each data point shows the theoretical prediction (*x*-coordinate) versus the experimental data (*y*-coordinate).

Therefore, we have added an SI figure (shown below in Figure S6, left side) that plots each theory-experiment data point in the same format we have used throughout our work. As noted by Reviewer 3 (see their Comment #5 below), the theoretical predictions lie closer to the experimental values if we allow a different avidity parameter c_{eff} for each influenza group (Figure S6, right side). This acknowledges that different influenza strains may have slightly different morphologies, which may alter the effective concentration of these multidomain antibodies. We have also added this plot to the SI to show the tradeoff between model complexity and the theory-experiment mismatch.

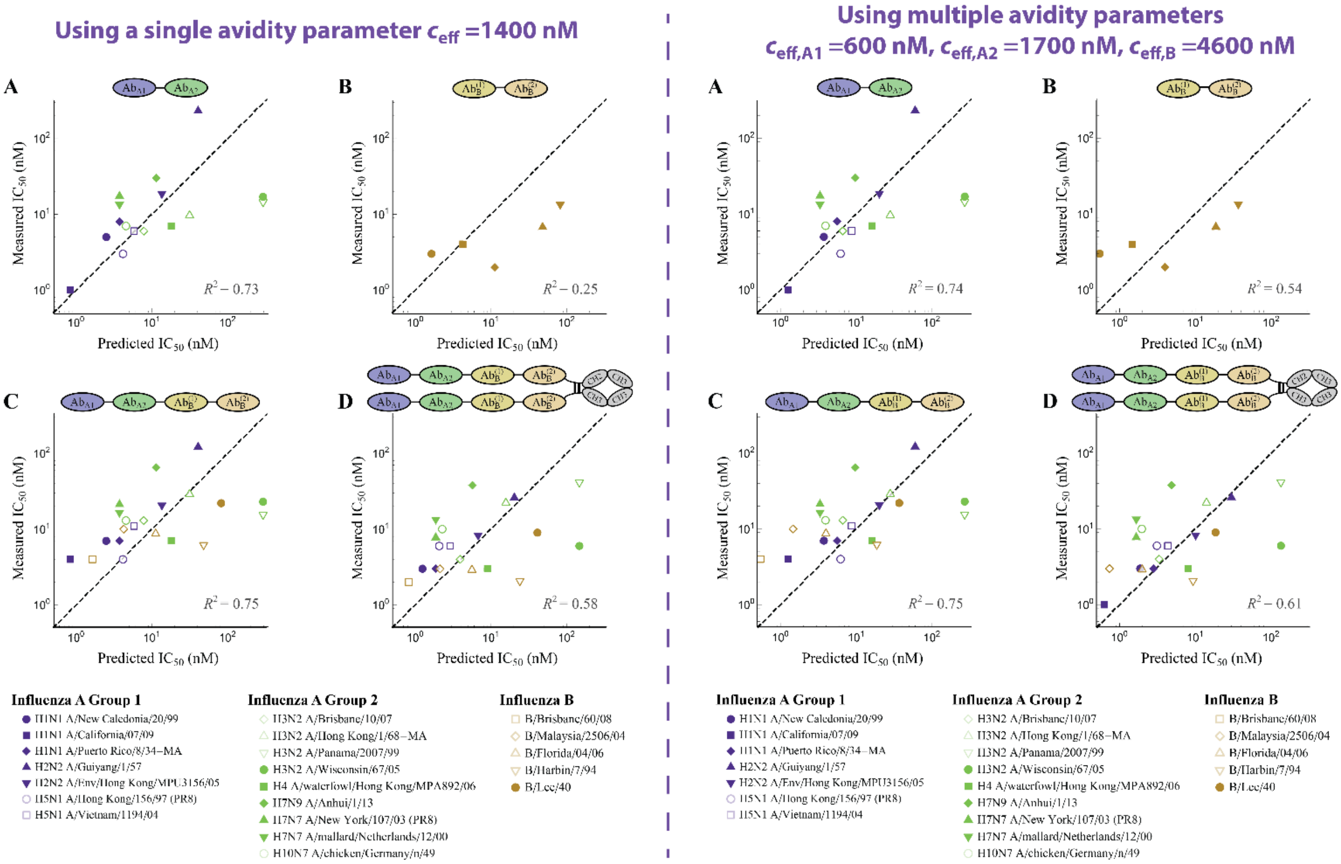

Figure S6. [Left panel] A breakdown of the multidomain influenza antibody data in Figure 5 of the main text (now Figure 6 in the new submission). [Right panel] This same breakdown using a different avidity parameter c_{eff} (inferred in Figure S12 of our Supporting Information file) for each influenza group.

Lastly, we compare our model to an uninformed model, which we define as a model that ignores the tethering component of the multidomain antibodies (i.e. the $c_{\text{eff}} \to 0$ limit). This independent model treats all four antibody domains as binding to independent sites (although Ab_{A1} and Ab_{A2} have no affinity for the influenza B strains

while $\mathrm{Ab}_\mathrm{B}^{(1)}$ and $\mathrm{Ab}_\mathrm{B}^{(2)}$ have no affinity to the influenza A1 or A2 strains). The results of this model are shown in Figure S7 below, demonstrating how without incorporating the boost in avidity, nearly all the resulting predictions are larger than the experimental measurements (i.e. the predictions are shifted to the right compared to the left panel of Figure S6 above).

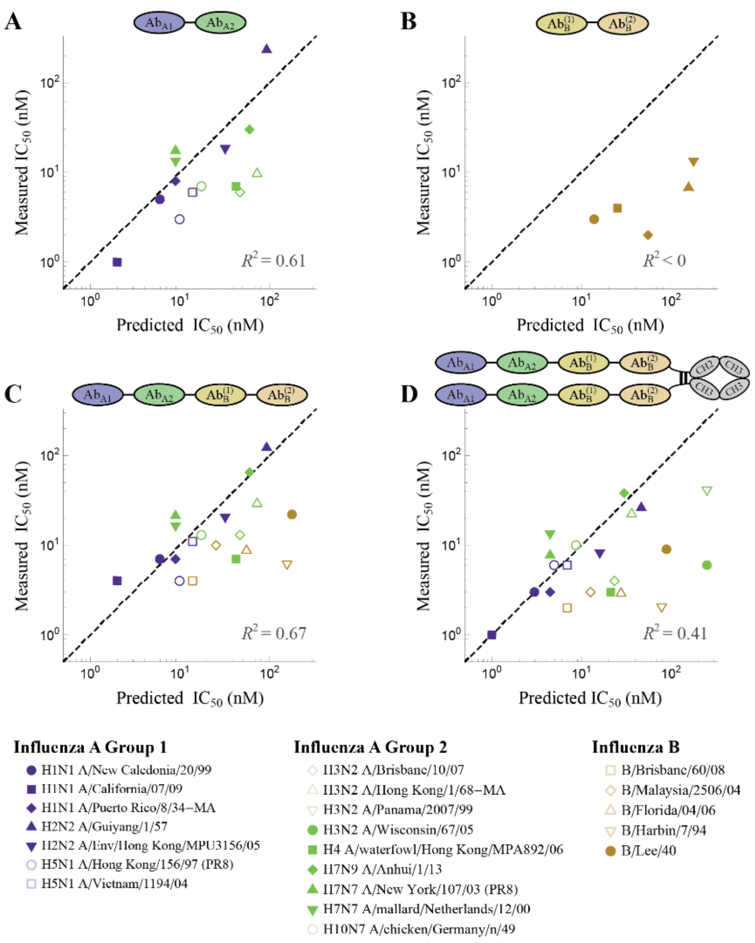

Figure S7. The multidomain influenza antibody data in Figure 5 of the main text compared to a model that ignores the multidomain antibody tethering and treats each domain as an independent antibody.

In summary, we have added Figures S6 and S7 as new SI sections (pages S15-S19). The former demonstrates how our model can be extended to more precisely characterize the data, whereas the latter provides context with which to compare our data to an uninformed model.

Reviewer 3

Motivated by the increasing popularity of combination antibody therapy, the authors developed an equilibrium binding model to describe the effect of an antibody mixture on the activity of a multi-epitope protein, and applied this model to published data sets on a cancer-related receptor and influenza viral proteins. By assuming either independent or exclusive binding of antibodies, the authors calculated the effect of antibody combinations, using measurements of individual antibodies and pairwise interactions or epitope mapping as inputs. They further considered engineered multi-domain antibodies and compared the predicted and measured potency.

My comments are as follows:

1. The authors claimed that 'the power of this model lies in its ability to make a large number of predictions based on a limited amount of data. For example, once 10 antibodies have been individually characterized, our model can predict how any of the $2^{10} = 1024$ combinations will behave.' This is not true, given that either pairwise measurements or information of epitope mapping has to be provided.

Author Reply

We thank the reviewer for their comments and address them one at a time:

The sentence referred to ("Once 10 antibodies have been individually characterized, our model can predict how any of the $2^{10} = 1024$ combinations will behave") is from our Author Summary. We agree that in addition to characterizing each antibody individually, we also require its epitope to predict all combinations. As noted by the reviewer, this epitope information can come from direct epitope mappings (e.g. crystallography, mutational analysis, pairwise competitive binding assay) or from the measurements of antibody mixtures (as shown in our Figure 3). We originally thought that this level of detail would be distracting in the Author Summary, but we agree that it is important to mention that epitope information is needed, and we have done so in the following revised version.

Old version (Page 1)

For example, once 10 antibodies have been individually characterized, our model can predict how any of the $2^{10} = 1024$ combinations will behave.

New version (Page 1)

For example, once 10 antibodies have been individually characterized and their epitopes have been mapped, our model can predict how any of the $2^{10} = 1024$ combinations will behave.

[Continued from above] More importantly, the assumption of either independent or exclusive binding seems not properly validated; e.g. as shown in Fig. 2A, prediction and measurement do not show a good match.

Author Reply

In Figure 2 of our main text (reproduced below in Figure S8), Panels A and B show the predictions using the distinct and overlapping epitopes inferred via SPR, whereas Panels C and D show the corresponding predictions if all antibodies either bind to purely distinct or purely overlapping epitopes, respectively.

There are two ways that we validated the SPR classifications of distinct versus overlapping epitopes. The first was by noting that in Figure S8, Panel B shows superior predictive power compared to Panels C and D. The second way that we validated the SPR classifications was to take the reverse approach and use the antibody mixture data to infer whether each antibody pair binds to an overlapping or distinct epitope (Figure 3 in our main text), which yielded a nearly identical epitope classification (only Ab_5 was misclassified, and the effects of its altered classification were very minor).

Within the context of our binding model (which neglects synergy), the agreement between these two methods suggests that the SPR classification is as good as possible. While we concur that the correspondence between the model predictions and experiments is not perfect, it nevertheless provides a quantitative framework to assess concepts such as synergy (discussed in the following point).

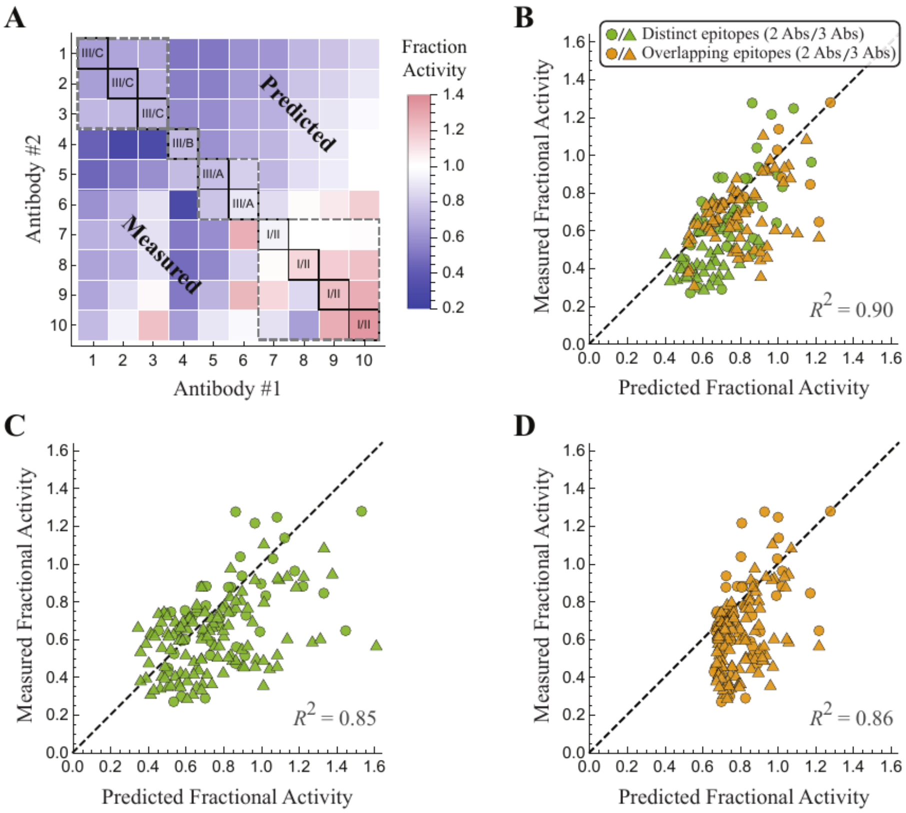

Figure S8. Predicting how antibody mixtures affect the epidermal growth factor receptor (EGFR). (A) The fractional activity of EGFR in the presence of monoclonal antibodies (diagonal) together with the measured (bottom-left) and predicted (top-right) activity of all 2-Ab combinations. The dashed gray boxes enclose antibody pairs that compete for the same epitope while all other pairs bind independently. (B) The predicted versus measured fractional activity for all 2-Ab (circles) and 3-Ab mixtures (triangles) using the same epitope mapping as in Panel A inferred by SPR. Without the epitope map, the activity of the mixtures could alternately be predicted by assuming that all antibodies either (C) bind independently or (D) compete for the same epitope; in either case, the resulting predictions fall further from the diagonal line, indicating poorer predictive power. This figure is a reproduction of Figure 2 from our main text.

[Continued from above] In addition, the measured activity is systematically lower than the predicted value. Both observations indicate potential importance of synergistic effect which is neglected in the analysis. Synergistic effects (e.g. through allostery) could be even more prominent in compliant protein molecules, which might restrict the generality of this model. This issue should be directly addressed.

Author Reply

We agree that the measured activity is systematically lower than the predicted activity, which is a strong indication that synergy does play a role in these antibody mixtures. To better quantify this effect, we have added the following SI plot (shown as Figure S9 below) which calculates the measured minus predicted activity for the data shown in Figure S8B. The asymmetry in the distribution to the left of zero may arise from synergistic interactions between antibodies. We have expanded our discussion of this synergistic effect to quantify the deviations between our model predictions and a "perfect" model where each antibody pair has an α_{eff} fit to the data. By comparing this "perfect" parameter value with the α value inferred from each individual mAb binding profile, we find that antibody mixtures have an average improvement of 10% in their potency over what would be expected from our binding model.

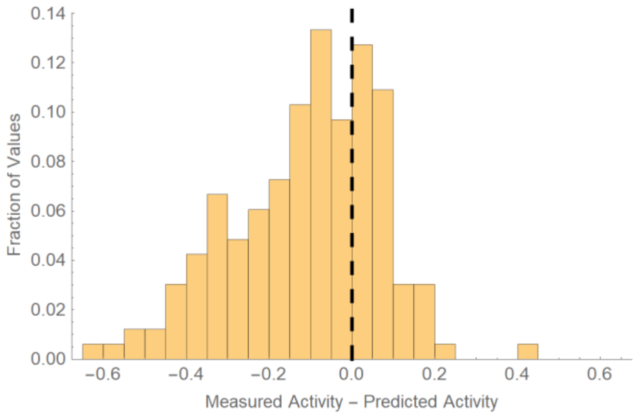

Figure S9. Subtracting the predicted EGFR activity from the measured activity for each antibody mixture in Figure 2B. A close match between theory and experiment would lie around zero (dashed vertical line), and the asymmetry of the distribution suggests that the model predictions are systematically larger than the experimental measurements, as may arise from synergistic interactions in antibody mixtures.

New version (Page 9)

The predictions incorporating the SPR mapping display a consistent bias towards having a slightly lower measured than predicted activity (Figure S9), suggesting that several pairs of antibodies enhance one another's binding affinity or potency. To quantify this effect, we recharacterized the activity from the 2-Ab mixtures using a synergistic model where each α_{eff} is fit to exactly match the data. We found an average value of $\frac{\alpha_{\text{eff}}}{\alpha} = 0.9$, showing that when pairs of antibodies are simultaneously bound, they typically boost their collective inhibitory activity by ~10%. This increase in the potency of antibody mixtures could arise from allosteric interactions where the binding of one antibody stabilizes a binding-favorable conformation for another antibody [Lee 2016, Howell 2017, Changeux 2012].

2. The authors stated that deviations from the predictions provide a rigorous way to measure antibody synergy, but no analysis is provided to support this statement. It would be more convincing to directly demonstrate this measure for the datasets used. This may offer a metric for the predictive power of this simplified model.

Author Reply

We believe that the previous point, where we now quantify the deviation of the potency parameter from the "perfect" model (demonstrating a 10% synergistic boost), addresses this point. We again thank the reviewer for raising this important issue.

3. 'This predictive power paves the way to expedite the design of future therapeutics'. This is a superfluous statement. I urge the authors to discuss in concrete terms what general design principles are learnt from this study.

Author Reply

We have tightened up this sentence as described below.

Old Version (Page 1)

This predictive power provides ample opportunities to test our model and paves the way to expedite the design of future therapeutics.

New Version (Page 1)

This predictive power can aid therapeutic efforts by assessing which combinations of antibodies will elicit the most effective response.

4. In Fig. 3A, it seems that the model fails to predict the epitope of most of the pairs, because two models predict very similar activities. And yet, this lack of epitope information doesn't impair the correlation between measured and predicted activity (Fig. 3B). Isn't this showing the model is not well constrained and lacking predictive power?

Author Reply

We thank the reviewer for noting this point of potential confusion. It is true that most antibody pairs have similar activities and cannot be directly categorized as binding to distinct or overlapping epitopes (hence they are shown as gray boxes in the top-right of Figure 3A, which we reproduce below as Figure S10). To circumvent this issue, we used the entire set of categorizations (i.e. all the pairwise classifications in the top-right of Figure S10) to determine the epitope groups.

Going through the data will make this procedure clear. Starting from the top-left, Ab_1, Ab_2, and Ab_3 all overlap with one another, and they did not explicitly overlap with any other antibodies (i.e. they had gray or green boxes with Ab_4-Ab_{10}). Hence, we assumed that Ab_1-Ab_3 bind to one epitope. Moving towards the bottom-right, Ab_4 and Ab_5 bind to an overlapping site and formed a second epitope group. Ab_6 did not explicitly overlap with any other antibody and was assumed to bind to its own third epitope group. Lastly, Ab_7-Ab_9 all overlapped with Ab_{10} (and thus by transitivity with each other), and hence these final four antibodies were assigned to a final epitope group. Note that in the absence of information, the default assumption is that each antibody binds to a distinct epitope.

In summary, the four epitope groups were: Ab_1-Ab_3, Ab_4-Ab_5, Ab_6, and Ab_7-Ab_{10}. Returning to the reviewer's question, it was these four antibody groups that were used in our model. For example, a mixture containing Ab_1 and Ab_4 used the distinct binding model because these two antibodies fall into different epitope groups (even though the direct categorization between these antibodies in Figure S10A was "Uncategorized"). To visually emphasize this point, we defined these four epitope groups using the dashed squares in Figure S10A.

Given this explanation, we believe that our model is both well constrained and has appreciable predictive power. Figures S8C and S8D above emphasize this point by showing how ignoring all information about the binding epitopes (and assuming that all antibodies either bind to distinct or overlapping epitopes) leads to worse characterizations of the data. In short, we have shown that activity data from antibody mixtures coupled with the distinct and overlapping binding models is sufficient to infer the antibody epitope groups and maintain the same level of predictive power as is gained through direct SPR binding competition assays.

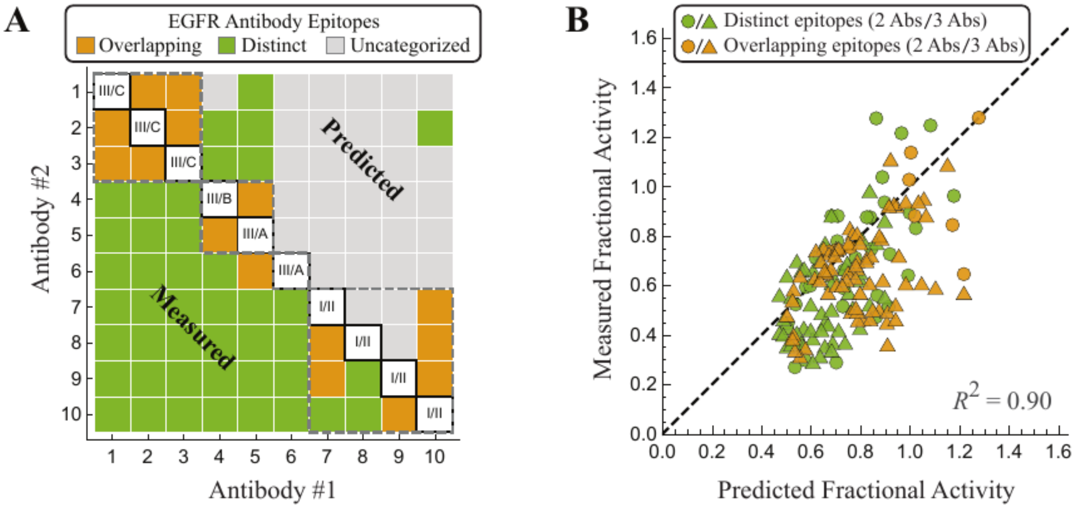

Figure S10. Classifying antibody epitopes as overlapping or distinct. (A) Comparing the experimentally measured activity to the overlapping or distinct epitope models enables us to characterize each antibody pair (provided the two models predict sufficiently different activities). The dashed squares represent the minimal epitope groupings inferred from this method. (B) The resulting predictions for the 2-Ab and 3-Ab mixtures have the same predictive power ($R^2 = 0.90$) as a model that relies on epitope groupings given by SPR measurements (Figure 2B).

We have made the following additions in the main text and in the Figure 3 caption to ensure that readers understand our categorization procedure.

Old Version (Page 10)
While the model predictions are much sparser (with the majority of antibody pairs uncategorized because the two model predictions were too close to one another), the classifications only disagreed with the SPR measurements in two cases (claiming that antibodies #7-8 overlap with antibody #10; notice that antibodies #7-8 have individual activities close to 1, making them difficult to characterize).

New Version (Page 10)
While the model predictions are much sparser (with the majority of antibody pairs uncategorized because the two model predictions were too close to one another), the classifications are nevertheless sufficient to group these antibodies by their epitopes. Antibodies #1-3 all overlap with one another (and do not explicitly overlap with any other antibodies) and hence are assumed to bind one epitope. Antibodies #4 and #5 overlap with each other and form a second epitope group. Antibody #6 did not explicitly overlap with any other antibody and forms a third epitope group. Lastly, Antibodies #7-9 all overlapped with #10, and hence these four antibodies bind to a fourth epitope. These four groups are shown by the dashed squares in Fig 3A, which only disagrees with the groups inferred by SPR (shown by the symbols along the white diagonal on each mAb) by claiming that Antibodies #4-5 (rather than #5-6) bind to an overlapping epitope.

These four epitope groups enable us to predict the activity of the 2-Ab and 3-Ab mixtures. Note that it is not the pairwise classification between two antibodies that determines whether we apply the distinct or competitive models, but rather the four groupings of antibody epitopes. For example, although antibodies #7 and #8 are uncategorized through their 2-Ab mixture, they fall within a single epitope group and hence are considered to bind competitively. Similarly, antibody #1 and #4 are modeled as binding independently because they belong to two distinct epitope groups.

Old Version (Page 11, Figure 3 Caption)
(A) Comparing the experimentally measured activity to the overlapping or distinct epitope models enables us to characterize each antibody pair (provided the two models predict sufficiently different activities).

New Version (Page 11, Figure 3 Caption)
(A) Comparing the experimentally measured activity to the overlapping or distinct epitope models enables us to characterize each antibody pair (provided the two models predict sufficiently different activities). The dashed squares represent the minimal epitope groupings inferred from this method.

5. The outliers in Fig. 5 are glaring. Analysis with synergistic effect should be added to show whether remedy can be achieved.

Author Reply

We agree that there are several points in Figure 5 (now Figure 6 in the new submission) whose predicted IC_{50} differs by more than 10-fold from the measured value, which is a marked discrepancy. Frankly, while we would love for the predictions to match the experimental data more closely, we are also intrigued by this discrepancy and hope that it will serve as a call-to-arms for groups looking to understand and harness synergistic effects. As such, we suggest that there is a place both for plots like our Figure 5 with glaring outliers as well as for the plots the Reviewer is suggesting that have additional synergy parameters and better resulting fits.

To briefly elaborate on this point, we call your attention to the $\mathrm{Ab}_{\mathrm{A1}}$-$\mathrm{Ab}_{\mathrm{A2}}$ pairs against the viral strains A/Panama/2007/99 and A/Wisconsin/67/05 discussed on page 13 (these strains are highlighted by the red lines in Figure 5A,C,D). These examples are especially fascinating because neither $\mathrm{Ab}_{\mathrm{A1}}$ alone, $\mathrm{Ab}_{\mathrm{A2}}$ alone, or the 1:1 *un*tethered mixture exhibited detectable neutralization against either viral strains ($\mathrm{IC}_{50} > 1000$ nM), but the tethered construct showed an $\mathrm{IC}_{50} \approx 15$ nM against both strains. This is a very curious phenomenon, and our initial thoughts were that tether may be involved in the neutralization or that these data points may simply be wrong. While we remain huge fans of the Laursen 2018 work, we believe that the straightforward analysis we conducted would have clearly revealed these outliers and led their team to investigate their data more deeply.

Returning to the Reviewer's point, our analysis already incorporates the spirit of synergistic parameters, since it includes the avidity term c_{eff} that quantifies the avidity boost of a tethered antibody. However, as discussed above in our response to Reviewer 2's Comment #5, we have now implemented separate c_{eff} parameters for the three influenza groups (A1, A2, and B), which has improved the theoretical predictions (especially for the influenza B strains). We feel that the resulting plots exhibit an excellent compromise between model complexity and predictive power.

6. As the author pointed out, SPR measurements indicate that there is a continuum of possible interactions, how this aspect shall be accounted for while still maintaining the simplicity and hence scalability of the model should be discussed in depth.

Author Reply

We thank the Reviewer for this point, which was also alluded to by Reviewer 2's Comment #2 above. As mentioned there, we have now added a new section to the main text and the new SI Section A.7 that quantifies the continuum of possible interactions between the distinct and overlapping binding models. We really like this new section and greatly appreciate this input.

7. The main text is hard to follow without frequently referring to the SI text. The Methods section in the main text should be expanded to include the essential information and formula for each subsection in Results.

Author Reply

We agree wholeheartedly with this point, and we have added several more sections to the Methods that now contain outlines of all the derivations and results in this work, with pointers to the relevant SI sections. Our Methods (pages 21-22) are now broken up into the following subsections, each containing more detailed descriptions:

- Models of EGFR antibody binding
- Models for influenza multidomain antibodies
- Goodness of fit
- Experimental data
